# Knowledge, Attitudes, and Practices Regarding Asthma in Children With Allergic Rhinitis

**DOI:** 10.1002/iid3.70277

**Published:** 2025-10-15

**Authors:** Benran Jiang, Ying Yuan, Wen Wen Koh, Junle Yan, Michelle Xiao Ying Law, Jian Shen

**Affiliations:** ^1^ Department of Pediatrics Parkway Shanghai Hospital Shanghai China; ^2^ Department of Pediatrics, Yueyang Hospital of Integrated Traditional Chinese and Western Medicine Shanghai University of Traditional Chinese Medicine Shanghai China

**Keywords:** allergic rhinitis, asthma, attitude, knowledge, parent, practice

## Abstract

**Background:**

Allergic rhinitis (AR) and asthma frequently coexist in children, with substantial overlap in pathophysiology and clinical management. Understanding caregivers' perspectives is important for improving disease control and outcomes. This study aimed to assess the knowledge, attitudes, and practices (KAP) of caregivers of children with AR toward associated asthma.

**Methods:**

A cross‐sectional survey was conducted in December 2023 at the pediatric outpatient clinic of an international hospital and two tertiary hospitals in Shanghai, China. Caregivers of children diagnosed with AR, rhinitis, asthma, or a history of wheezing were invited to complete the survey.

**Results:**

A total of 460 participants completed the questionnaire, of whom 316 (68.7%) were female. The median (25th percentile, 75th percentile) scores for knowledge, attitude, and practice were 9 (6, 11), 24 (22, 26), and 34 (32, 38), respectively. Knowledge was positively correlated with attitude (*r* = 0.386, *p* < 0.001) and with practice, while attitude was also positively correlated with practice. Multivariate analysis showed that having a master's degree or above was independently associated with better knowledge, whereas nonmedical occupation and absence or uncertainty of family history were independently associated with worse knowledge. Having a male child and a child aged ≥ 3 years were independently associated with more positive practice, while uncertainty about family history was independently associated with more negative practice.

**Conclusion:**

Caregivers of children with AR demonstrated adequate knowledge, moderate attitude, and moderate practice toward asthma. Targeted educational interventions are recommended for caregivers with lower educational levels, nonmedical occupations, and uncertainty about family history.

## Summary

- This study examined the KAP of caregivers of children with allergic rhinitis regarding associated asthma.
- It identified important gaps in understanding, particularly among caregivers with lower education and nonmedical backgrounds.
- The findings suggest that targeted educational interventions could improve asthma management, ultimately benefiting child health outcomes.
- For readers of the journal, this research underscores the importance of caregiver education in pediatric asthma management and provides evidence that may inform clinical practice and public health strategies.

## 1 Introduction

In contemporary clinical research, increasing attention has been given to the link between allergic rhinitis (AR) and asthma, particularly in children. AR, a common respiratory condition triggered by allergens such as pollen and dust mites, presents with symptoms ranging from nasal congestion to headaches [1, 2]. Asthma, characterized by chronic airway inflammation, remains a major global health issue, affecting approximately 235 million people worldwide [3, 4]. Epidemiological evidence shows that children with AR are more likely to develop asthma [5, 6]. These conditions represent atopic diseases, with 30% of patients with AR also having asthma, and 70% of patients with asthma presenting with AR [7]. This close association highlights their interconnected nature within the spectrum of atopic disorders. Globally, studies have demonstrated varying levels of caregiver knowledge and practices regarding pediatric asthma management. Research from developed countries indicates that although caregivers generally possess basic knowledge about asthma, significant gaps remain in their understanding of the relationship between AR and asthma. Caregivers also show differences in their recognition of asthma triggers, management strategies, and the unified airway concept linking AR and asthma [5, 6]. Regional variations have been reported, with differences in overall knowledge scores and management practices across populations [8, 9].

In Asia, limited research has examined caregivers' perspectives on the AR–asthma relationship. Most studies have focused on asthma or AR individually rather than exploring their interconnection. In China, research has investigated parental knowledge of individual respiratory conditions, but there is a lack of comprehensive studies assessing the knowledge, attitudes, and practices (KAP) of caregivers regarding the AR–asthma link in pediatric populations.

The KAP theory is widely used to explain health behaviors [10]. It has been applied in healthcare research to assess understanding and behaviors related to various medical conditions [11, 12]. Although the connection between AR and asthma is well established, existing KAP studies have largely examined these diseases in isolation, with few exploring the dual burden of AR and asthma, particularly in Asian populations. Given their prevalence in children, it is important to investigate caregivers' perceptions, attitudes, and practices to understand how these conditions are managed. Such research provides insights into parental behaviors and decision‐making, offering evidence to improve healthcare delivery. To date, no studies in China have specifically evaluated the KAP of caregivers regarding AR and asthma. This study aimed to fill this knowledge gap by examining the challenges and needs faced by caregivers of children with these conditions. Accordingly, this study was designed to investigate the KAP of caregivers of children with AR, particularly in relation to asthma. The objective was to address the research gap by exploring how caregivers perceive, understand, and manage the interplay between AR and asthma. The findings are intended to provide a foundation for developing targeted health education programs that improve caregiver awareness, thereby strengthening their ability to manage and prevent AR and asthma in children.

## 2 Methods

This cross‐sectional study was conducted at the pediatric outpatient clinics of an international hospital and two tertiary hospitals in Shanghai, China: Parkway Shanghai Hospital, Yueyang Hospital of Integrated Traditional Chinese and Western Medicine affiliated with Shanghai University of Traditional Chinese Medicine, and Shanghai Sixth People's Hospital affiliated with Shanghai Jiao Tong University. The study was carried out from December 2023 to January 2024, during the winter season. A non‐probability convenience sampling method was used to recruit eligible participants during outpatient visits. Ethical approval was obtained from the Parkway Shanghai Hospital Medical Ethics Committee (PSH‐ETHICS‐FO‐001). Written informed consent was obtained from all participants before enrollment.

### 2.1 Inclusion and Exclusion Criteria

Parents or caregivers of children with AR, rhinitis, asthma, or a history of wheezing who visited the pediatric outpatient clinic in December 2023 were invited to participate. Exclusion criteria included incomplete surveys, a completion time of less than 60 s, or responses indicating uniform selection of the same option across all items in the knowledge, attitude, and practice sections.

### 2.2 Sample Size Calculation

The sample size was determined using the standard formula for cross‐sectional studies:

$$n=\frac{Z^{2}P(1-P)}{d^{2}}$$

where:

*Z* represents the *z*‐score at a 95% confidence level, corresponding to 1.96. This value is the critical threshold for a two‐sided 95% confidence interval in a standard normal distribution, where 2.5% lies in each tail (cumulative probability = 0.975). The value was obtained from a standard *Z*‐distribution table.

*P* denotes the estimated proportion of the population, set at 50% (0.5) to yield the maximum sample size. *d* is the margin of error, defined as 0.05 (5%).

Based on these values, the sample size was recalculated. Using the conservative assumption that *P* = 0.5, *d* = 0.05, and a 95% confidence level, the required sample size was 384.16. For practical purposes, this was rounded up to 385 participants to ensure adequate statistical power.

### 2.3 Questionnaire Design

A 34‐item closed‐ended questionnaire was developed to assess the KAP of caregivers of children with AR toward associated asthma. It was modified and validated by three pediatric respiratory and allergy experts, followed by a pilot test with 30 participants. The test yielded a reliability coefficient of 0.958 and a Kaiser–Meyer–Olkin (KMO) value of 0.913. The final questionnaire, administered in Chinese, consisted of four domains: (i) sociodemographic characteristics of children and caregivers, (ii) knowledge, (iii) attitude, and (iv) practice regarding AR and asthma. Sociodemographic variables included the child's age and gender, caregiver's age, gender, highest educational level, occupation, relationship with the child, and family history. Domain scores were calculated based on item responses. The knowledge domain contained 11 items, each with three options: “true,” “false,” and “uncertain.” A correct answer was scored as 1, and incorrect or “uncertain” responses were scored as 0. Knowledge scores ranged from 0 to 11, with higher scores indicating better knowledge. The attitude domain (six items) and practice domain (eight items) were assessed using a Likert scale ranging from 1 (*strongly agree*) to 5 (*strongly disagree*). Responses were scored from 5 (positive) to 0 (negative). Attitude scores ranged from 6 to 30, and practice scores from 8 to 40, with higher scores reflecting more positive attitudes or practices. Items that could not be assigned scores were analyzed as categorical variables (see Supporting Information S2: Questionnaire).

### 2.4 Study Procedures

Parents or caregivers of children with AR, rhinitis, asthma, or a history of wheezing who attended pediatric outpatient clinics were invited to participate. Once they provided consent, the survey was administered during their visits. The questionnaire was designed using a professional online survey platform (Wenjuanxing, www.wjx.cn). Distribution was facilitated through the WeChat social media platform. Participants could complete the survey by scanning a WeChat QR code to access the Wenjuanxing Mini Program or by manually completing a paper‐based questionnaire. Informed consent was obtained before participation.

A total of 523 surveys and consent forms were completed. To ensure validity, surveys that appeared randomly filled were excluded. Exclusion criteria included completion time under 60 s or identical responses across all knowledge, attitude, and practice domains. Of the 523 surveys, 24 were excluded for completion time under 60 s and 39 for identical responses. The final analysis included 460 valid questionnaires.

### 2.5 Statistical Analysis

Domain scores were first tested for normality. For normally distributed data, results were expressed as mean ± standard deviation, while non‐normally distributed data were presented as median (25th percentile, 75th percentile). Demographic characteristics and item responses were described as *N* (%). Comparisons of domain scores across demographic groups were made using *t*‐tests for normally distributed continuous variables and the Wilcoxon–Mann–Whitney test for non‐normally distributed variables. For comparisons across three or more groups, ANOVA or Kruskal–Wallis tests were applied. To assess interrelationships among KAP, Pearson's correlation coefficient was used for normally distributed data, and Spearman's rank correlation for non‐normal data. Further analyses employed univariate and multivariate regression, incorporating variables with significance levels of *p* < 0.1 and *p* < 0.25.

## 3 Results

### 3.1 Demographic Characteristics of Participants and KAP Scores

As shown in Table 1, 334 participants (72.6%) were older than 35 years, 316 (68.7%) were female, and 352 (76.5%) were parents. Among the children, 249 (54.1%) were male and 389 (84.6%) were older than 3 years. About one‐third of the children (32.6%, *n* = 150) had a family history of the disease, and nearly two‐thirds (63.3%, *n* = 291) had a history of AR. The median (25th percentile, 75th percentile) scores for knowledge, attitude, and practice were 9 (6, 11), 24 (22, 26), and 34 (32, 38), respectively. Analyses of demographic variables showed that knowledge scores differed by education level (*p* = 0.005), occupation (*p* < 0.001), relationship with the child (*p* = 0.017), and family history (*p* < 0.001). Practice scores varied according to caregiver gender (*p* = 0.023), child gender (*p* = 0.023), child age (*p* < 0.001), occupation (*p* = 0.048), and family history (*p* = 0.021).

**Table 1:** Baseline table.

	*N* (%)	Knowledge (K)		Attitude (A)		Practice (P)	
		Median (25th percentile, 75th percentile)	*p*	Median (25th percentile, 75th percentile)	*p*	Median (25th percentile, 75th percentile)	*p*
*Total*	460	9 (6, 11)		24 (22, 26)		34 (32, 38)	
*Age*			0.638		0.072		0.248
≤ 35 years	126 (27.4)	9 (6, 10)		24 (22, 27)		34 (32, 39)	
> 35 years	334 (72.6)	9 (6, 11)		24 (22, 26)		34 (32, 37)	
*Gender*			0.130		0.304		0.023
Female	316 (68.7)	9 (7, 10)		24 (22, 26)		34 (32, 38.5)	
Male	144 (31.3)	9 (5, 11)		24 (21, 26)		33 (32, 37.5)	
*Child's gender*			0.509		0.258		0.031
Female	211 (45.9)	9 (6, 10)		24 (22, 26)		33 (32, 37)	
Male	249 (54.1)	9 (6, 11)		24 (22, 26)		34 (32, 39)	
*Child's age*			0.081		0.079		< 0.001
≤ 3 years	71 (15.4)	8 (2, 11)		24 (21, 25)		32 (30, 36)	
> 3 years	389 (84.6)	9 (7, 10)		24 (22, 26)		34 (32, 39)	
*Education*			0.005		0.584		0.116
College and below	109 (23.7)	8 (4, 10)		24 (22, 26)		33 (32, 37)	
Bachelor's	247 (53.7)	9 (7, 10)		24 (22, 26)		34 (32, 39)	
Master's and above	104 (22.6)	10 (7, 11)		24 (22, 26)		34 (32, 38)	
*Occupation*			< 0.001		0.140		0.048
Medical industry practitioner	82 (17.8)	10 (9, 11)		24.5 (23, 26)		34 (32, 38)	
Government employee	56 (12.2)	8 (5, 10)		24 (21, 26)		32 (31, 36.5)	
Private/foreign‐funded enterprise employee	151 (32.8)	9 (7, 11)		24 (22, 26)		35 (32, 39)	
Other	171 (37.2)	8 (5, 10)		24 (22, 26)		34 (32, 38)	
*Caregiver–child relationship*			0.017		0.182		0.178
Parents	352 (76.5)	9 (6.5, 11)		24 (22, 26)		34 (32, 38)	
Grandparents	33 (7.2)	8 (3, 10)		23 (20, 26)		33 (31, 37)	
Other	75 (16.3)	9 (6, 10)		24 (21, 26)		33 (32, 37)	
*Family history of disease*			< 0.001		0.165		0.021
Yes	150 (32.6)	10 (8, 11)		24 (22, 26)		35 (32, 39)	
No	231 (50.2)	9 (6, 10)		24 (21, 26)		34 (32, 38)	
Uncertain	79 (17.2)	8 (3, 10)		24 (22, 26)		33 (32, 36)	

8. Does your child have the following medical history	*N* (%)
History of allergic rhinitis	291 (63.3)
History of rhinitis	100 (21.7)
History of asthma	74 (16.1)
History of wheezing	34 (7.4)
Uncertain	91 (19.8)

As shown in Table 1, 334 participants (72.6%) were older than 35 years, 316 (68As shown in Supporting Information S1: Table 1, the median scores for knowledge, attitude, and practice were 9, 24, and 34, respectively, with 43.4%, 40.9%, and 45.7% of participants scoring above these values.

### 3.2 Distribution of Responses for KAP

**Figure 1:**
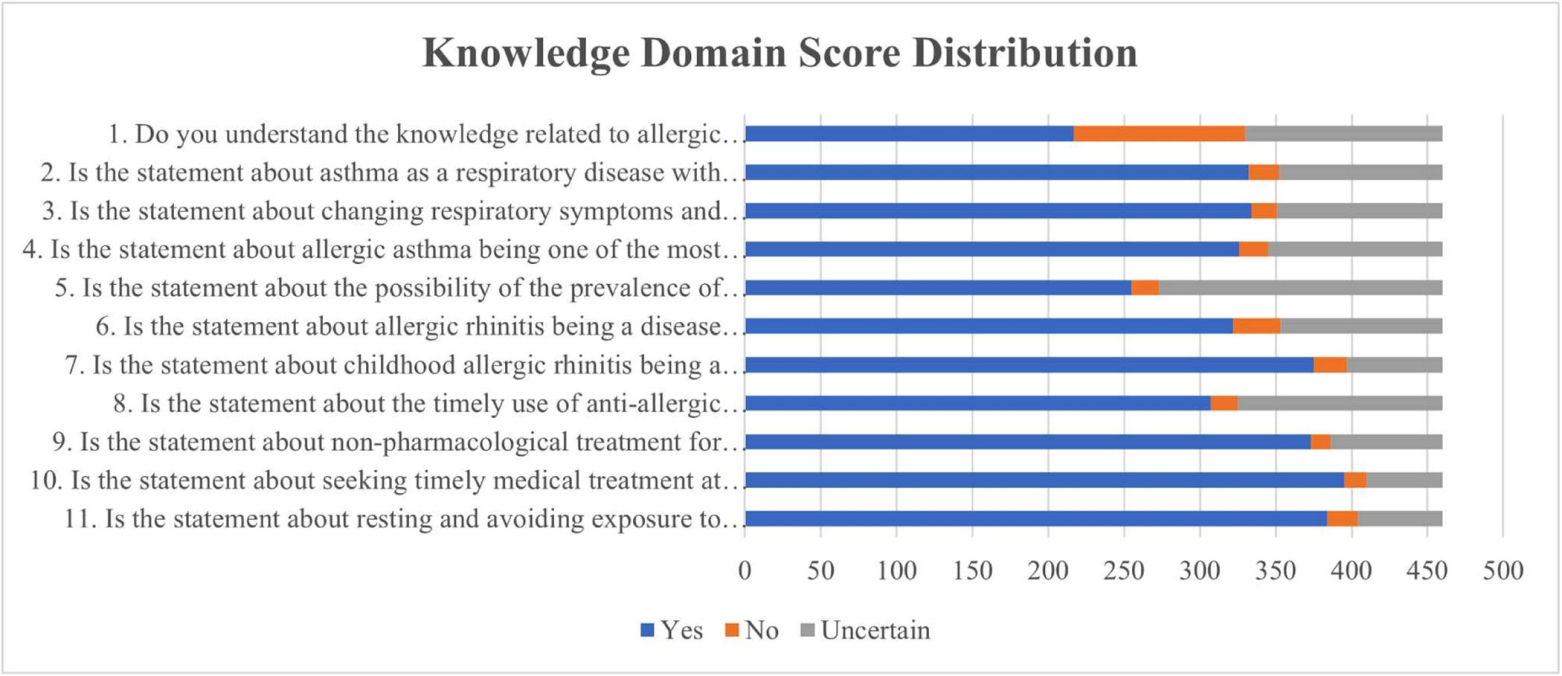
Knowledge domain score distribution.

As shown in Figure 1, in the knowledge domain, the two highest correct response rates were 85.9% for the item “Seeking timely medical treatment at a pediatric pulmonology clinic when a child exhibits symptoms of allergic rhinitis‐associated asthma is correct” (K10) and 83.5% for the item “Resting and avoiding exposure to dry, cold air helps prevent worsening of symptoms such as nasal congestion and wheezing in children with allergic rhinitis‐associated asthma” (K11). The lowest correct response rates were 47.2% for “Do you understand the knowledge related to allergic rhinitis‐associated asthma?” (K1) and 55.4% for “The prevalence of childhood asthma in China may be higher than the currently expected level” (K5).

Table 2 shows that 47.0% strongly agreed that integrated medical intervention for childhood asthma should also be applied to AR (A2). In addition, 39.3% strongly agreed that avoiding allergen exposure, removing triggers, controlling AR, and nutritional support are important to prevent asthma attacks (A5). Furthermore, 46.3% strongly recognized the value of protective measures against allergens and appropriate exercise for AR‐associated asthma (A6). Concern about the development of AR in children with asthma was reported by 44.8% of participants (A1). However, 48.9% and 54.8% gave neutral responses regarding the priority of glucocorticoid administration (A3) and its clinical benefit (A4).

**Table 2:** Attitude domain score distribution.

	a. Very worried	b. Worried	c. Neutral	d. Not worried	e. Not worried at all
1. If your child has a history of asthma, are you worried about the occurrence of allergic rhinitis in your child?	184 (40.0)	206 (44.8)	59 (12.8)	4 (0.9)	7 (1.5)

	a. Strongly agree	b. Agree	c. Neutral	d. Disagree	e. Strongly disagree
2. Do you agree that integrated medical intervention for childhood asthma should also be used for treating allergic rhinitis?	216 (47.0)	156 (33.9)	83 (18.0)	3 (0.7)	2 (0.4)
3. Do you agree that corticosteroids are the preferred medication for controlling and preventing the recurrence of asthma attacks?	78 (17.0)	120 (26.1)	225 (48.9)	32 (7.0)	5 (1.1)
4. Do you think the clinical treatment of corticosteroids for childhood allergic asthma has more benefits than drawbacks?	62 (13.5)	125 (27.2)	252 (54.8)	17 (3.7)	4 (0.9)
5. Do you agree that, besides clinical drug control to relieve asthma, measures such as avoiding allergens, removing triggering factors, controlling allergic rhinitis, and nutritional support are equally important to prevent asthma attacks?	181 (39.3)	164 (35.7)	109 (23.7)	4 (0.9)	2 (0.4)
6. Do you think routine protective measures such as avoiding allergens and engaging in appropriate exercise are crucial for allergic rhinitis‐associated asthma?	213 (46.3)	171 (37.2)	69 (15.0)	5 (1.1)	2 (0.4)

Participants generally demonstrated proactive practices (Table 3). To prevent asthma, 55.4% encouraged their children to exercise properly (P1), 53.9% avoided allergens in daily routines (P2), and 41.7% increased nasal cleaning for their children (P3). In addition, 52.4% were willing to take their children for allergy testing (P5), and 48.3% were willing for their children to avoid allergens once identified through testing (P6).

**Table 3:** Practice domain score distribution.

	a. Strongly willing to	b. Willing to	c. Neutral	d. Unwilling to	e. Strongly unwilling to
1. Are you willing to encourage your child to exercise appropriately within an acceptable range to enhance physical fitness and prevent asthma?	255 (55.4)	167 (36.3)	35 (7.6)	1 (0.2)	2 (0.4)
2. Are you willing to pay attention to avoiding allergens in lifestyle, hygiene, and diet to prevent your child from developing asthma?	248 (53.9)	178 (38.7)	29 (6.3)	3 (0.7)	2 (0.4)
3. Are you willing to enhance cleaning of your child's nasal cavity to prevent asthma triggered by allergic rhinitis?	180 (39.1)	192 (41.7)	76 (16.5)	10 (2.2)	2 (0.4)
4. Are you willing to proactively learn or acquire background knowledge related to allergic rhinitis‐associated asthma?	176 (38.3)	223 (48.5)	55 (12.0)	4 (0.9)	2 (0.4)
5. Are you willing to take your child to the hospital for allergy tests?	128 (27.8)	241 (52.4)	82 (17.8)	7 (1.5)	2 (0.4)
6. After identifying allergens, are you willing to actively help your child avoid exposure to them?	186 (40.4)	222 (48.3)	42 (9.1)	6 (1.3)	4 (0.9)
7. After your child is diagnosed and receives medical treatment, are you willing to spend time addressing your child's psychological issues (rejection of treatment drugs, curiosity leading to attempts to contact allergens, etc.) and guide your child psychologically regarding the pros and cons?	175 (38.0)	241 (52.4)	41 (8.9)	2 (0.4)	1 (0.2)
8. During your child's treatment process, are you willing to communicate with the doctor in a timely manner about the progress of the condition, and discuss specific treatment plans based on your child's actual situation and psychological state?	207 (45.0)	224 (48.7)	26 (5.7)	1 (0.2)	2 (0.4)

### 3.3 Factors Associated With KAP

As shown in Supporting Information S1: Table 2, knowledge was positively correlated with attitude (*r* = 0.386, *p* < 0.001) and with practice (*r* = 0.287, *p* < 0.001). Attitude was also positively correlated with practice (*r* = 0.561, *p* < 0.001).

A stepwise forward approach was applied, and variables were entered into the multivariate regression model at *p* < 0.1. As shown in Table 4, education at the master's level or above was independently associated with better knowledge (OR = 1.942, 95% CI: 1.072–3.518, *p* = 0.029). Nonmedical occupation and absence or uncertainty of family history were independently associated with poorer knowledge (OR < 1, *p* < 0.005). Having a male child (OR = 1.472, 95% CI: 1.005–2.158, *p* = 0.047) and having a child older than 3 years (OR = 2.515, 95% CI: 1.424–4.441, *p* = 0.001) were independently associated with more positive practice. Uncertainty about family history was independently associated with more negative practice (OR = 0.522, 95% CI: 0.294–0.925, *p* = 0.026). No factor was independently associated with attitude.

**Table 4:** Statistical analysis (univariate, multivariate analysis).

4.1 Knowledge domain							
Cutoff value: > 9/≤ 9		Univariate		Multivariate (*p* < 0.1)		Multivariate (*p* < 0.25)	
	No.	OR (95% CI)	*p*	OR (95% CI)	*p*	OR (95% CI)	*p*
*Age*							
≤ 35 years	51/126	ref.					
> 35 years	148/334	1.170 (0.772, 1.774)	0.459				
*Gender*							
Female	137/316	ref.					
Male	62/144	0.988 (0.664, 1.471)	0.952				
*Child's gender*							
Female	88/211	ref.					
Male	111/249	1.124 (0.776, 1.629)	0.536				
*Child's age*							
≤ 3 years	28/71	ref.					
> 3 years	171/389	1.205 (0.719, 2.019)	0.480				
*Education*							
College and below	37/109	ref.		ref.		ref.	
Bachelor's	106/247	1.463 (0.915, 2.340)	0.112	1.346 (0.821, 2.207)	0.239	1.346 (0.821, 2.207)	0.239
Master's and above	56/104	2.270 (1.306, 3.947)	0.004	1.942 (1.072, 3.518)	0.029	1.942 (1.072, 3.518)	0.029
*Occupation*							
Medical industry practitioner	53/82	ref.		ref.		ref.	
Government employee	18/56	0.259 (0.126, 0.533)	< 0.001	0.248 (0.118, 0.520)	< 0.001	0.248 (0.118, 0.520)	< 0.001
Private/foreign‐funded enterprise employee	69/151	0.460 (0.264, 0.802)	0.006	0.498 (0.282, 0.880)	0.016	0.498 (0.282, 0.880)	0.016
Other	59/171	0.288 (0.166, 0.500)	< 0.001	0.342 (0.192, 0.610)	< 0.001	0.342 (0.192, 0.610)	< 0.001
*Caregiver–child relationship*							
Parents	163/352	ref.					
Grandparents	10/33	0.504 (0.233, 1.090)	0.082				
Other	26/75	0.615 (0.366, 1.035)	0.067				
*Family history of disease*							
Yes	83/150	ref.		ref.		ref.	
No	92/231	0.534 (0.352, 0.810)	0.003	0.489 (0.316, 0.757)	0.001	0.489 (0.316, 0.757)	0.001
Uncertain	24/79	0.352 (0.198, 0.628)	< 0.001	0.351 (0.194, 0.636)	0.001	0.351 (0.194, 0.636)	0.001

4.2 Attitude domain							
Cutoff value: > 24/≤ 24		Univariate		Multivariate (*p* < 0.1)		Multivariate (*p* < 0.25)	
	No.	OR (95% CI)	*p*	OR (95% CI)	*p*	OR (95% CI)	*p*
*Age*							
≤ 35 years	59/126	ref.				ref.	
> 35 years	129/334	0.715 (0.473, 1.081)	0.111			0.534 (0.334, 0.853)	0.009
*Gender*							
Female	136/316	ref.					
Male	52/144	0.748 (0.498, 1.123)	0.162				
*Child's gender*							
Female	81/211	ref.					
Male	107/249	1.209 (0.832, 1.758)	0.319				
*Child's age*							
≤ 3 years	22/71	ref.		ref.		ref.	
> 3 years	166/389	1.658 (0.965, 2.850)	0.067	1.658 (0.965, 2.850)	0.067	2.336 (1.272, 4.292)	0.006
*Education*							
College and below	39/109	ref.					
Bachelor's	106/247	1.349 (0.847, 2.150)	0.207				
Master's and above	43/104	1.265 (0.728, 2.199)	0.404				
*Occupation*							
Medical industry practitioner	41/82	ref.					
Government employee	19/56	0.514 (0.254, 1.037)	0.063				
Private/foreign‐funded enterprise employee	60/151	0.659 (0.384, 1.134)	0.132				
Other	68/171	0.660 (0.388, 1.122)	0.125				
*Caregiver–child relationship*							
Parents	148/352	ref.					
Grandparents	11/33	0.689 (0.324, 1.465)	0.333				
Other	29/75	0.869 (0.521, 1.448)	0.590				
*Family history of disease*							
Yes	66/150	ref.					
No	94/231	0.873 (0.576, 1.323)	0.523				
Uncertain	28/79	0.699 (0.398, 1.226)	0.212				

4.3 Practice domain							
Cutoff value: > 34/≤ 34		Univariate		Multivariate (*p* < 0.1)		Multivariate (*p* < 0.25)	
	No.	OR (95% CI)	*p*	OR (95% CI)	*p*	OR (95% CI)	*p*
*Age*							
≤ 35 years	62/126	ref.					
> 35 years	148/334	0.821 (0.545, 1.238)	0.348				
*Gender*							
Female	154/316	ref.		ref.		ref.	
Male	56/144	0.669 (0.448, 1.000)	0.050	0.705 (0.466, 1.067)	0.098	0.705 (0.466, 1.067)	0.098
*Child's gender*							
Female	86/211	ref.		ref.		ref.	
Male	124/249	1.442 (0.996, 2.088)	0.053	1.472 (1.005, 2.158)	0.047	1.472 (1.005, 2.158)	0.047
*Child's age*							
≤ 3 years	19/71	ref.		ref.		ref.	
> 3 years	191/389	2.640 (1.505, 4.630)	0.001	2.515 (1.424, 4.441)	0.001	2.515 (1.424, 4.441)	0.001
*Education*							
College and below	42/109	ref.					
Bachelor's	119/247	1.483 (0.937, 2.348)	0.093				
Master's and above	49/104	1.421 (0.824, 2.451)	0.206				
*Occupation*							
Medical industry practitioner	37/82	ref.					
Government employee	18/56	0.576 (0.283, 1.171)	0.128				
Private/foreign‐funded enterprise employee	76/151	1.232 (0.719, 2.114)	0.448				
Other	79/171	1.044 (0.615, 1.772)	0.872				
*Caregiver–child relationship*							
Parents	167/352	ref.					
Grandparents	13/33	0.720 (0.347, 1.493)	0.377				
Other	30/75	0.739 (0.445, 1.226)	0.241				
*Family history of disease*							
Yes	79/150	ref.		ref.		ref.	
No	102/231	0.711 (0.470, 1.073)	0.105	0.718 (0.470, 1.099)	0.127	0.718 (0.470, 1.099)	0.127
Uncertain	29/79	0.521 (0.298, 0.911)	0.022	0.522 (0.294, 0.925)	0.026	0.522 (0.294, 0.925)	0.026

## 4 Discussion

Caregivers of children with AR demonstrated sufficient knowledge and moderate attitudes and practices toward asthma. The positive correlations between knowledge, attitude, and practice highlight the interconnected nature of these elements within the parental perspective. Healthcare providers are advised to develop targeted educational interventions to enhance knowledge and positively influence attitudes and practices, particularly among caregivers with lower education levels and those without a medical background.

The study showed that caregivers of children with AR possessed satisfactory knowledge, maintained moderate attitudes, and displayed appropriate practices in asthma management. The median scores for knowledge, attitude, and practice reflected a balanced understanding and engagement with the subject. These findings are consistent with existing literature on parental knowledge and behaviors related to childhood asthma [12, 13]. The positive correlations observed between knowledge, attitude, and practice align with previous studies, emphasizing their interconnected role in shaping health‐related behaviors [14, 15].

Analysis of demographic characteristics revealed significant variations in knowledge and practice scores according to education, occupation, relationship with the child, and family history. These findings are consistent with research showing that socioeconomic factors and family history are strongly associated with parental understanding and practices in managing childhood asthma [16, 17]. Gender‐related differences in knowledge and practice scores add further nuance, suggesting that tailored educational strategies may be required for specific demographic groups [18, 19].

Multivariate analysis identified factors independently associated with knowledge and practice. Higher education levels (master's and above) were positively associated with better knowledge, consistent with previous studies highlighting the role of education in health literacy [20, 21]. In contrast, nonmedical occupation and lack of or uncertainty about family history were negatively associated with knowledge, underscoring the need for targeted interventions in these groups [22].

Independent factors associated with positive practices included having a male child and having an older child. These associations provide useful insights for tailoring interventions to parental circumstances. Conversely, uncertainty about family history was negatively associated with practice, highlighting the importance of effective communication and awareness campaigns that address familial health information [23, 24, 25]. No factor was independently associated with attitude, suggesting that determinants of parental attitudes toward asthma management may be more complex and require further investigation.

The key findings provide a comprehensive view of caregivers' KAP toward asthma associated with AR in children. Within the knowledge domain, notable gaps were observed, including uncertainty regarding the prevalence of childhood asthma in China and the familial tendency of AR. These findings are consistent with prior research demonstrating gaps in caregivers' knowledge of pediatric respiratory conditions [8].

In the attitude domain, caregivers expressed concerns about the occurrence of AR in their children with asthma, reflecting the interconnected nature of these respiratory conditions. Diverse attitudes toward medication use, particularly corticosteroid treatment, with varied levels of agreement on its benefits and drawbacks, further highlight the nuanced perspectives held by caregivers. These findings are consistent with studies that emphasize the complex decision‐making process caregivers face in managing childhood asthma [26, 27].

The practice domain demonstrated caregivers' willingness to engage in health‐promoting behaviors. Many caregivers expressed positive attitudes toward encouraging physical activity and avoiding allergens, yet notable differences were observed in their willingness to undertake specific actions, such as conducting allergy tests or addressing psychological aspects of treatment. These results align with existing literature, which highlights the multifaceted challenges caregivers encounter in implementing preventive measures and adhering to treatment plans [6, 28].

When compared with existing studies, the findings were compatible with broader literature on caregiver KAP related to pediatric asthma. Similar knowledge gaps and diverse attitudes toward treatment have been reported in different cultural and healthcare settings [9]. Variations in willingness to adopt specific practices also reflect the complex interplay of cultural, psychological, and practical factors in caregiver decision‐making [29]. To address these gaps and variations in caregivers' KAP, targeted strategies should be developed. Educational interventions should aim to improve caregivers' understanding of key issues, including the prevalence of childhood asthma, familial tendencies, and the importance of timely medical treatment. Such interventions can be delivered through culturally appropriate and accessible materials, supported by healthcare providers or community‐based initiatives [17].

Given the varied attitudes toward medication use observed among caregivers, it is essential for healthcare providers to strengthen the dissemination of scientific knowledge. This can be achieved through multiple approaches, such as organizing educational lectures, publishing accessible science‐based articles, and distributing informative materials in healthcare settings. These efforts should provide clear, evidence‐based information on the management of AR and associated asthma, emphasizing both the benefits and potential risks of available treatments. Integrating psychological support into asthma management is also crucial, with interventions designed to address caregivers' concerns about treatment rejection and curiosity‐driven exposure attempts [30]. In terms of practices, interventions should focus on enhancing caregivers' readiness to engage in actions such as conducting allergy tests and addressing psychological aspects of treatment. Practical guidance, hands‐on training, and continuous support from healthcare professionals can help strengthen caregivers' confidence and competence in carrying out these practices [13].

Despite the valuable insights provided by this study, several limitations should be noted. First, the cross‐sectional design limits the ability to establish causality, offering only a snapshot rather than a longitudinal perspective. Second, reliance on self‐reported data introduces the possibility of recall bias, which may affect the accuracy of participants' responses. Third, the study was conducted in one international hospital and two tertiary hospitals in Shanghai, which may restrict the generalizability of the findings to broader populations. Furthermore, as the survey was conducted in winter, a season often associated with increased allergic symptoms, caregivers may have been more alert and proactive, potentially introducing seasonal bias. Nonetheless, the strengths of this study include its robust sample size, comprehensive assessment of the KAP domains, and the application of multivariate analysis to identify key associations. These features contribute to a more nuanced understanding of caregivers' KAP regarding asthma in children with AR, despite the inherent limitations of the study design and data collection methods.

In conclusion, caregivers of children with AR demonstrated sufficient knowledge, with moderate attitudes and practices toward asthma. Healthcare professionals should prioritize targeted educational interventions, especially for caregivers with lower educational attainment or nonmedical occupations. Emphasizing the importance of awareness of family medical history may also play a critical role in improving knowledge, which could in turn foster more positive attitudes and practices. Incorporating these approaches into routine healthcare interactions may enable caregivers to manage childhood asthma more effectively.

## Author Contributions

Benran Jiang conducted the study, conceptualized and designed the study, collected data, performed statistical analysis, interpreted the findings, and drafted the manuscript. Ying Yuan contributed to data interpretation and drafting of the manuscript. Wen Wen Koh, Junle Yan, and Michelle Xiao Ying Law participated in data collection and statistical analysis. Jian Shen contributed to study conceptualization and design and critically reviewed the manuscript. All authors read and approved the final manuscript.

## Ethics Statement

All methods were performed in accordance with relevant guidelines. This study was conducted in accordance with the Declaration of Helsinki (2000) of the World Medical Association. Approval was obtained from the Parkway Shanghai Hospital Medical Ethics Committee (PSH‐ETHICS‐FO‐001). Informed consent was obtained from all participants before their involvement.

## Consent

All participants provided electronic informed consent before completing the online survey. Consent was specifically obtained for the anonymized data to be used and published.

## Conflicts of Interest

The authors declare no conflicts of interest.

## Supporting information


**Table S1:** Score Distribution. **Table S2:** Correlation Analysis

Supporting Information.

## Data Availability

All data generated or analyzed in this study are included in this published article.
