# Supplementary material for: Knowledge, Attitudes, and Practices Regarding Asthma in Children With Allergic Rhinitis
Source: Immun Inflamm Dis. 2025 Oct 15;13(10):e70277. doi: 10.1002/iid3.70277 (PMC12521876; doi:10.1002/iid3.70277)
Supplement: Supplementary file 1 — Table S1: Score Distribution. Table S2: Correlation Analysis [file IID3-13-e70277-s002.doc]

**Table S1**: Score Distribution

|  | **Median** | **25th Percentile** | **75th Percentile** | **Minimum** | **Maximum** | **≤Median N(%)** | **＞Median N(%)** |
| --- | --- | --- | --- | --- | --- | --- | --- |
| **Knowledge Domain** | 9 | 6 | 11 | 0 | 11 | 261(56.7) | 199(43.3) |
| **Attitude Domain** | 24 | 22 | 26 | 6 | 30 | 272(59.1) | 188(40.9) |
| **Practice Domain** | 34 | 32 | 38 | 8 | 40 | 250(54.3) | 210(45.7) |

**Table S2: Correlation Analysis**

|  | **Knowledge** | **Attitude** | **Practice** |
| --- | --- | --- | --- |
| **Knowledge** | 1.000 | 0.386(P < 0.001) | 0.287(P < 0.001) |
| **Attitude** | 0.386(P < 0.001) | 1.000 | 0.561(P < 0.001) |
| **Practice** | 0.287(P < 0.001) | 0.561(P < 0.001) | 1.000 |
