# Supplementary material for: Knowledge, Attitudes, and Practices Regarding Asthma in Children With Allergic Rhinitis
Source: Immun Inflamm Dis. 2025 Oct 15;13(10):e70277. doi: 10.1002/iid3.70277 (PMC12521876; doi:10.1002/iid3.70277)
Supplement: Supplementary file 2 — Supporting Information. [file IID3-13-e70277-s001.doc]

Questionnaire Number:

| Dear Participant,  We are researchers from pediatric outpatient clinic of an international hospital, and we sincerely invite you to participate in our study. This research aims to understand parents' knowledge, attitudes, and treatment practices regarding childhood allergic rhinitis-related asthma. The findings will provide a basis for developing scientific early intervention strategies, which may help improve patients' health in the future. Your participation in this study is entirely voluntary. If you agree to participate, please refer to the following instructions:  Complete the Questionnaire: The questionnaire has no right or wrong answers. Please fill it out based on your actual situation. If you have any questions during the process, feel free to ask us. Once completed, please submit it promptly.  Confidentiality and Privacy: This study involves a simple questionnaire and will not harm your physical or psychological health. However, it does involve some privacy issues, such as your gender, age, etc. We will strictly maintain the confidentiality of your information, so please feel free to provide accurate responses.  Participant Rights: As a participant, you can inquire about any information related to the study and its progress at any time. If you decide to withdraw from the study, please notify us, and your data will not be included in the research results.  Lastly, we sincerely thank you for taking the time to support our scientific research amidst your busy schedule  □ I acknowledge and consent to the use of the data collected for scientific research.  Informed Consent Signature:  Date of Participation: |
| --- |

**Part 1 Basic Information**

**Age**

1. ≤35 years
2. ＞35 years

**Gender**

1. Female
2. Male

**Child's Gender**

1. Female
2. Male

**Child's Age**

1. ≤3 years
2. ＞3 years

**Education**

1. College and below
2. Bachelor’s
3. Master’s and above

**Occupation**

1. Medical industry practitioner
2. Government employee
3. Private/foreign-funded enterprise employee
4. Other

**Caregiver-Child Relationship**

1. Parents
2. Grandparents
3. Other

**Does your child have the following medical history :**

History of allergic rhinitis

History of rhinitis

History of asthma

History of wheezing

Uncertain

**Family history of disease**

1. Yes
2. No
3. Uncertain

**Part II: Understanding Asthma Associated with Allergic Rhinitis**

1. **Do you understand the knowledge related to allergic rhinitis-associated asthma?**

A. Yes B. No C. Uncertain

1. **Is the statement about asthma as a respiratory disease with chronic airway inflammation and increased airway reactivity, often exacerbating at night or in the early morning, correct?**

A. Yes B. No C. Uncertain

1. **Is the statement about changing respiratory symptoms and severity during asthma attacks, often accompanied by obstructive ventilatory dysfunction, correct?**

A. Yes B. No C. Uncertain

1. **Is the statement about allergic asthma being one of the most common phenotypes, especially in childhood asthma, often associated with inflammatory diseases such as allergic rhinitis, correct?**

A. Yes B. No C. Uncertain

1. **Is the statement about the possibility of the prevalence of childhood asthma in China being higher than the current expected level, correct?**

A. Yes B. No C. Uncertain

1. **Is the statement about allergic rhinitis being a disease influenced by both genetic and environmental factors, showing a familial tendency, correct?**

A. Yes B. No C. Uncertain

1. **Is the statement about childhood allergic rhinitis being a common chronic disease with main symptoms including itchy nose, sneezing, clear nasal discharge, and nasal congestion, different from a cold, correct?**

A. Yes B. No C. Uncertain

1. **Is the statement about the timely use of anti-allergic treatment for childhood allergic rhinitis-associated asthma, including antihistamines like loratadine and cetirizine, and leukotriene receptor antagonists like montelukast, along with the use of corticosteroid nasal sprays externally, correct?**

A. Yes B. No C. Uncertain

1. **Is the statement about non-pharmacological treatment for childhood allergic rhinitis, such as nasal irrigation under the guidance of a doctor to remove allergens and secretions, helping to relieve symptoms like nasal congestion, itching, and sneezing, correct?**

A. Yes B. No C. Uncertain

1. **Is the statement about seeking timely medical treatment at a regular respiratory clinic when a child exhibits symptoms of allergic rhinitis-associated asthma, correct?**

A. Yes B. No C. Uncertain

1. **Is the statement about resting and avoiding exposure to dry, cold air to prevent worsening of symptoms such as nasal congestion and wheezing when a child has symptoms of allergic rhinitis-associated asthma, correct?**

A. Yes B. No C. Uncertain

**Part III Attitudes towards asthma associated with allergic rhinitis**

**1. If your child has a history of asthma, are you worried about the occurrence of allergic rhinitis in your child?**

a. Very Worried b. Worried c. Neutral d. Not Worried e. Not Worried at all

1. **Do you agree that integrated medical intervention for childhood asthma should also be used for treating allergic rhinitis?**

a. Strongly Agree b. Agree c. Neutral d. Disagree e. Strongly Disagree

1. **Do you agree that corticosteroids are the preferred medication for controlling and preventing the recurrence of asthma attacks?**

a. Strongly Agree b. Agree c. Neutral d. Disagree e. Strongly Disagree

1. **Do you think the clinical treatment of corticosteroids for childhood allergic asthma has more benefits than drawbacks?**

a. Strongly Agree b. Agree c. Neutral d. Disagree e. Strongly Disagree

1. **Do you agree that, besides clinical drug control to relieve asthma, measures such as avoiding allergens, removing triggering factors, controlling allergic rhinitis, and nutritional support are equally important to prevent asthma attacks?**

a. Strongly Agree b. Agree c. Neutral d. Disagree e. Strongly Disagree

**6. Do you think routine protective measures such as avoiding allergens and engaging in appropriate exercise are crucial for allergic rhinitis-associated asthma?**

a. Strongly Agree b. Agree c. Neutral d. Disagree e. Strongly Disagree

**Part IV Practice of Asthma Behaviors Associated with Allergic Rhinitis**

1. **Are you willing to encourage your child to exercise appropriately within an acceptable range to enhance physical fitness and prevent asthma?**

a. Strongly Willing to b. Willing to c. Neutral d. Unwilling to e. Strongly Unwilling to

1. **Are you willing to pay attention to avoiding allergens in lifestyle, hygiene, and diet to prevent your child from developing asthma?**

a. Strongly Willing to b. Willing to c. Neutral d. Unwilling to e. Strongly Unwilling to

1. **Are you willing to enhance cleaning of your child's nasal cavity to prevent asthma triggered by allergic rhinitis?**

a. Strongly Willing to b. Willing to c. Neutral d. Unwilling to e. Strongly Unwilling to

1. **Are you willing to proactively learn or acquire background knowledge related to allergic rhinitis-associated asthma?**

a. Strongly Willing to b. Willing to c. Neutral d. Unwilling to e. Strongly Unwilling to

1. **Are you willing to take your child to the hospital for allergy tests?**

a. Strongly Willing to b. Willing to c. Neutral d. Unwilling to e. Strongly Unwilling to

1. **After identifying allergens, are you willing to actively help your child avoid exposure to them?**

a. Strongly Willing to b. Willing to c. Neutral d. Unwilling to e. Strongly Unwilling to

1. **After your child is diagnosed and receives medical treatment, are you willing to spend time addressing your child's psychological issues (rejection of treatment drugs, curiosity leading to attempts to contact allergens, etc.) and guide your child psychologically regarding the pros and cons?**

a. Strongly Willing to b. Willing to c. Neutral d. Unwilling to e. Strongly Unwilling to

**8. During your child's treatment process, are you willing to communicate with the doctor in a timely manner about the progress of the condition, and discuss specific treatment plans based on your child's actual situation and psychological state?**

a. Strongly Willing to b. Willing to c. Neutral d. Unwilling to e. Strongly Unwilling to

**This concludes the questionnaire, thank you again for filling out the questionnaire and supporting children's health!**
